# Multi-teacher based knowledge distillation for retinal vessel segmentation

**DOI:** 10.1007/s13755-025-00356-4

**Published:** 2025-06-19

**Authors:** Abdullah Eid, Musa Aydin, Zeki Kuş

**Affiliations:** Department of Computer Engineering, Fatih Sultan Mehmet Vakif University, Beyoğlu, 34450 Istanbul Türkiye

**Keywords:** Knowledge Distillation, Retinal Vessel Segmentation, Multi Teacher Learning, Medical Imaging

## Abstract

Accurate segmentation of retinal vessels is crucial for the early diagnosis and management of various ocular diseases. Existing methods often struggle to segment thin vessels, leading to missed diagnoses and inaccurate treatment plans. This study proposes a novel Multi-Teacher Based Knowledge Distillation (MTKD) method for Retinal Vessel Segmentation (RVS) to address this challenge. Our approach utilizes the expertise of multiple teacher networks, each specialized in learning different vessel characteristics. Specifically, we train three distinct teacher networks: one on the original ground truth, one on a modified ground truth highlighting thin vessels, and another on a modified ground truth emphasizing thick vessels. The student network is then trained to minimize the knowledge discrepancy between its predictions and the soft predictions of all three teachers. By incorporating knowledge from these specialized teachers, the student network effectively learns to segment both thin and thick vessels with improved accuracy. We evaluate our method on two retinal fundus image datasets and two angiography datasets, demonstrating highly competitive performance compared to state-of-the-art methods. The proposed method improves the baseline U-Net model by up to 8.44 points in F1 and 10.42 points in IOU. Additionally, we introduce a penalization technique to the student model’s loss function, further enhancing segmentation performance. Comprehensive ablation studies validate the effectiveness of the multi-teacher approach, the choice of loss functions, and the impact of model complexity. Our findings suggest that MTKD offers a promising approach for enhancing the robustness and accuracy of RVS. All source code, datasets, and results are made publicly available to support reproducibility and further research.

## Introduction

Retinal vessel segmentation (RVS) is the process of automatically extracting blood vessels from digital retina images. This process is crucial for determining the vascular structure of the retina and is vital for the early identification of retinal diseases. It is especially important for identifying conditions such as diabetic retinopathy, glaucoma, hypertensive retinopathy, and vascular occlusion. RVS can assist doctors in making diagnoses and improve patient monitoring [1]. Despite its importance, RVS is inherently challenging due to the thin and complex nature of the vascular structures, which are often found in low-contrast areas. Additionally, noise, reflections, and other retinal structures can further complicate the task, making manual segmentation time-consuming and requiring a high level of expertise.

U-Net [2] is a convolutional neural network model designed for segmenting medical images. The model’s distinctive U-shaped structure allows it to extract features from input images across several layers and generate output images from these features. U-Net is widely utilized for RVS, and numerous studies have proposed architectural variations to tackle specific challenges in this field. The Dual Encoding U-Net (DEU-Net) model [3] for RVS uses two encoders–spatial and context paths–along with a feature fusion module and channel attention mechanism to combine and prioritize features. The Modified Residual U-Net (MResU-Net) model introduces a novel residual block structure with batch normalization before activation units and dropout layers to improve performance and convergence and prevent overfitting [4]. SCS-Net [5] introduces a scale and context-sensitive network that addresses challenges like scale variations and complex anatomical contexts. Zhou et al. [6] enhance the robustness of RVS models trained on noisy or incomplete labels with a Study Group Learning (SGL), while Liu et al. [7] propose a Full-Resolution Network (FRN) combined with a Dual-Threshold Iteration (DTI) strategy to improve segmentation accuracy. Sun et al. propose the SDAU-Net [8] model, introducing series deformable convolution (SDC) to replace standard convolution. This improvement enhances feature extraction for vessels with varying curvatures. Additionally, the model incorporates lightweight (LAM) and dual attention mechanisms (DAM) to enhance middle-layer feature utilization, thereby reducing small vessel loss and mis-segmentation. Moreover, recent studies have suggested different approaches to improve the segmentation efficiency of U-Net models for RVS [9–12]. Shin et al. [13] propose a hybrid CNN-GNN architecture designed to enhance vessel segmentation accuracy by jointly learning local appearance and global vessel connectivity. This approach models the graphical structure of vessel neighborhoods, leading to better performance in identifying vessels. Yan et al. [14] address the discrepancies in segmentation accuracy between thick and thin vessels by introducing a three-stage model. This model segments thick and thin vessels separately before integrating the results, thereby improving the accuracy for thin vessels. Vessel-Net [15] presents a lightweight architecture that incorporates inception-residual blocks alongside multi-path supervision strategies, achieving state-of-the-art results in retinal vessel segmentation tasks. HAnet [16] proposes a multi-decoder architecture that differentiates between challenging and straightforward segmentation regions. Attention mechanisms enhance feature learning in difficult areas, such as thin or uncertain vessels. To address the challenge of domain discrepancy in cross-modality vessel contour detection, Yihua et al. [17] introduce a bilateral domain adaptation framework that aligns shared and private label spaces across IVUS and OCT modalities.

Knowledge Distillation (KD) is a technique where a quicker and smaller model, called the"student model,"learns from a larger and more intricate model, named the"teacher model."In this technique, the outputs from the teacher model guide the student, with the goal of enhancing the student’s ability to generalize and perform well. Furthermore, the teacher and student models can have a similar architecture, which can lead to better results than using the teacher model alone. This alignment allows the student model to efficiently apply the knowledge acquired from the teacher [18–21]. Multi-teacher KD is a method that transfers knowledge from various teacher models to one student model. In this method, each teacher model provides learning from a different perspective, and this combined knowledge improves the performance of the student model. Multi-teacher KD is effective for datasets with diverse features and for multitasking learning scenarios. In medical image segmentation, studies like Multi-teacher Cross-modal Knowledge Distillation (MTCM-KD) for brain tumor segmentation [22] and M-MTUNet for rectal cancer segmentation [23] have demonstrated the efficacy of teacher-based KD frameworks in transferring diverse knowledge to student models. For the segmentation of retinal vessels, KD has been examined via several novel methodologies. Gu et al. [24] introduce a two-stage framework to enhance vessel segmentation using self-distillation and texture enhancements, performing well on DRIVE and CHASEDB1 datasets. Similarly,"Lesion-aware Knowledge Distillation (LKD)"[25] employs intra and inter-image knowledge transfer to improve diabetic retinopathy segmentation. In the teacher-based KD context, the study proposes an unsupervised multi-target domain adaptation (MTDA) framework [26]. In addition, UKnow-Net [27] introduces a knowledge-enhanced U-Net architecture for RVS. It leverages multi-teacher KD to integrate domain-specific expertise from teacher models trained on DRIVE, CHASEDB1, DCA1, and CHUAC datasets. This approach significantly improves generalization and outperforms existing methods in sensitivity, specificity, F1 score, and IoU metrics.

Our motivation seeks to capture diverse aspects of vessel morphology, as standard models often struggle to segment thick and thin vessels uniformly. By utilizing a multi-teacher knowledge distillation approach, where each teacher model specializes in different vessel characteristics, we aim to consolidate this expertise into a singular, efficient student model. This model leverages the specialized knowledge from multiple teachers, enhancing generalization and segmentation accuracy for all vessel sizes, particularly thin vessels, without requiring multiple complex models. We introduce a novel multi-teacher KD method for RVS conducted on the DRIVE, CHASEDB1, DCA1, and CHUAC. The ground truth images in the datasets are manually divided into three categories: thin vessels, thick vessels, and original vessels. Three separate teacher models are trained for these ground truths, and the knowledge from these models is combined and transferred to a student model using a response-based multi-teacher KD approach. Compared to U-Net-based approaches and other methods in the literature, the proposed method achieved highly competitive performance, delivering state-of-the-art results on certain datasets and excelling in the segmentation of both thin and thick vessels. We build upon the advancements in RVS using KD methods while addressing limitations observed in existing literature. Below, we highlight how our approach differs from previous works and contributes uniquely to the field: **(1) Categorization of Vessel Types:** While existing methods like UKnow-Net [27] use KD to learn features across entire datasets, our study uniquely categorizes vessels into three distinct types: thin, thick, and original vessels. By training separate models for each vessel type, we improve the segmentation accuracy of fine vessels, which are typically harder to detect and prone to misclassification. **(2) Response-Based Multi-Teacher Knowledge Distillation:** The multi-teacher KD approach in"UKnow-Net"[27] uses pseudo-labels generated by teacher models for knowledge transfer. Similarly,"Lesion-aware Knowledge Distillation (LKD)"[25] employs intra- and inter-image distillation for lesion-specific segmentation. Our method differs by introducing a response-based multi-teacher KD mechanism that aggregates knowledge from specialized teacher models (trained on thin, thick, and original vessels) and effectively transfers this aggregated knowledge to a unified student model. This strategy ensures that the student model generalizes better across diverse datasets while maintaining high sensitivity to refined vessel structures. **(3) Specialized Knowledge Transfer for Vessel Types:** Unlike Guo et al. [24], which enhances vessel segmentation through texture and resolution improvements, our approach directly focuses on vessel type-specific learning. This distinction helps the student model distinguish between vessel types more effectively and enhances the segmentation of challenging thin vessels. **(4) Comprehensive Ablation Studies:** Differing from other studies, we have performed various ablation studies to understand and comprehensively discuss the proposed method’s key components. These studies provide valuable for understanding the efficacy of different loss functions, the balance between model complexity and performance, the distinct roles of teacher models, the architectural sensitivity of the student model, and the impact of the temperature parameter in KD.

The primary novelty and most significant contribution of this work lies in designing and applying a response-based multi-teacher knowledge distillation (MTKD) framework. This method is specifically tailored to enhance the segmentation of challenging vascular structures, particularly thin vessels, across diverse medical imaging modalities, such as retinal fundus and coronary imaging. We also highlight its main contributions as follows:

- **Novel Multi-Teacher Knowledge Distillation Framework for Enhanced Vessel Segmentation:** We introduce a response-based multi-teacher knowledge distillation (MTKD) framework for improved retinal and coronary vessel segmentation. This framework uniquely trains multiple specialized teacher networks (e.g., focused on original, thin, and thick vessel structures), effectively distilling their diverse expertise into a single, efficient student model. A key component of this framework is a novel loss penalization technique applied to the student model, further refining segmentation accuracy, particularly for challenging fine vessel details. The study also analyzes the computational efficiency of the proposed method by comparing the number of parameters, FLOPs, and parameter size of the original student model with a Lite student model. This analysis emphasizes the balance between model size and performance.
- **Comprehensive Experimental Validation and In-depth Analysis on Diverse Medical Datasets:** The efficacy of the proposed MTKD framework is rigorously demonstrated through extensive evaluations on four publicly available datasets (DRIVE, CHASEDB1, DCA1, and CHUAC), which include different imaging modalities (retinal fundus and coronary angiography). Our method shows significant performance improvements over existing state-of-the-art techniques. This validation is supported by comprehensive ablation studies that meticulously analyze the impact of various components, including different loss functions, student model complexity (including a"Lite"version for computational efficiency analysis), individual teacher contributions, student architecture sensitivity, and knowledge distillation temperature. These studies provide deep insights into the framework’s behavior and justify our design choices.
- **Publicly Available Resources:** The study makes all the source code, input images, manually annotated thin and thick ground truths, and original ground truths publicly available, promoting reproducibility and further research in the field.

This study is structured as follows: Section"Methods"outlines the methodology used in the research. Section"Experimental Design"presents the experimental design, including datasets, implementation details, and evaluation metrics. Section"Results and Discussion"provides and discusses the results for the proposed method. Finally, Section"Conclusion"concludes the study and suggests areas for future research.

## Methods

**Fig. 1:**
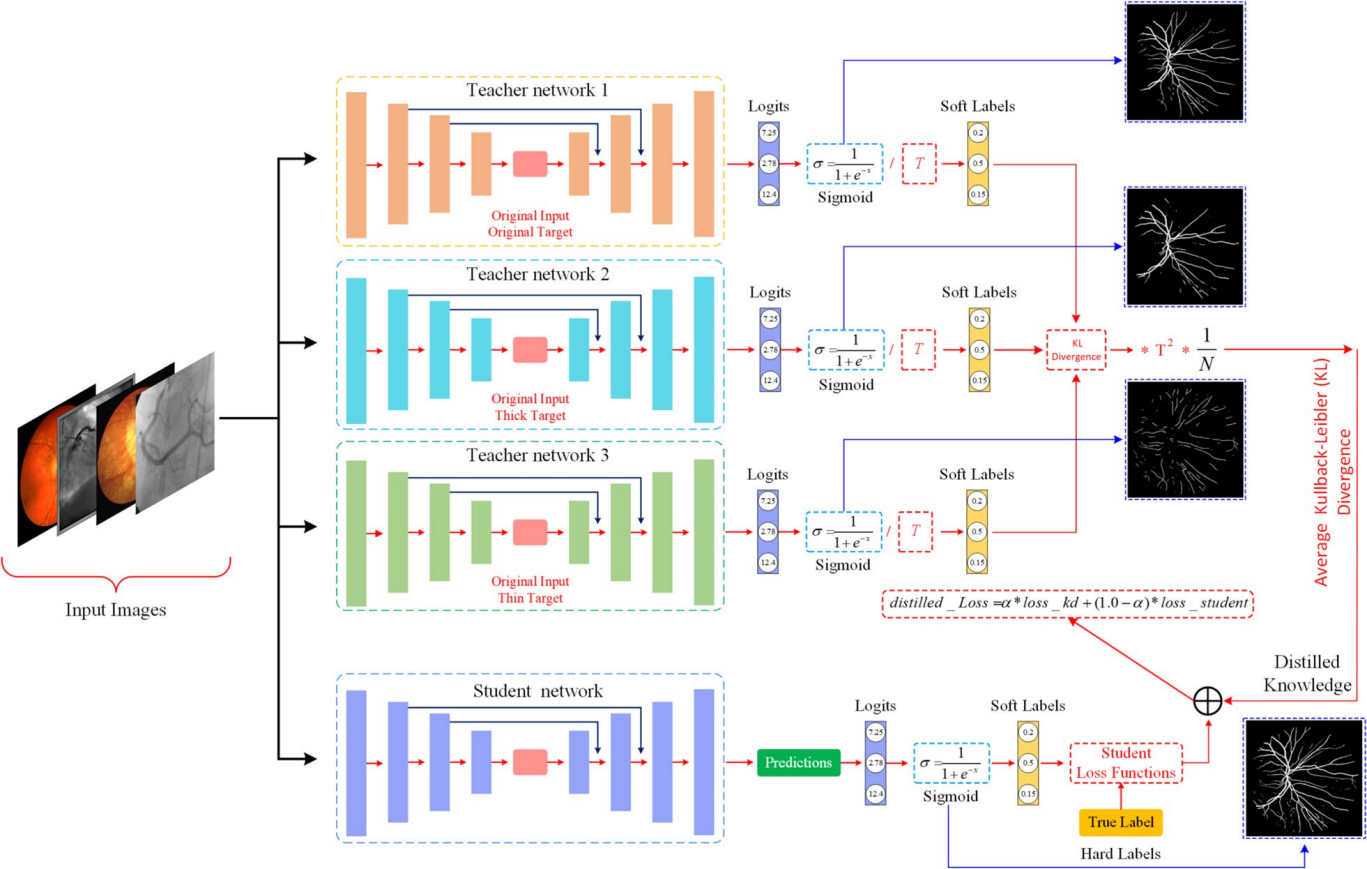
Proposed system overview: Multi-teacher KD medical image segmentation network model architecture. *T *indicates the temperature parameter. Student loss functions indicate the losses used in experimental studies. In experimental studies, the error of the student network is calculated with 8 different loss functions, these are DiceLoss, CombinedLoss, TverskyLoss, ComboLoss, SoftDiceLoss, DiceBCELoss, FocalLoss, FocalTverskyLoss, respectively. Logits are the network’s raw outputs, while soft labels are the values obtained by applying an activation function to these outputs. True-hard labels refer to the binary ground truth segmentations. *loss*_*kd:* Knowledge Distillation Loss – Measures how well the student model matches the outputs (i.e. soft labels) of the teacher model. *loss*_*student:* Measures how well the student model matches the hard labels. $\alpha :$ This is the hyperparameter that weights the two loss terms. In all experiments in this study, $\alpha$ was determined as 0.5 so that the loss values calculated from each teacher network and the loss value calculated from the student network contribute equally

In this study, we propose the Multi-Teacher-Based Knowledge Distillation (MTKD) approach, which focuses on improving the high-accuracy segmentation of thin vessels to address the challenges of vessel segmentation in medical images. The MTKD approach involves training multiple teacher networks, each focused on a different type of vessel segmentation. These networks then share their expertise (distill knowledge) with one unified student network. The MTKD strategy entails training several teacher networks, each focused on a distinct type of vessel segmentation. The proposed method is novel and aims to utilize multiple teacher networks to enhance the learning process of a student network focused on segmenting retinal vessels. In the proposed study, we present an innovative approach called response-based KD [18] to focus on learning the knowledge from the last output layer of the pre-trained teacher model as a response. Response-based knowledge distillation seeks to enhance the similarity of predictions between the teacher and the student. The proposed approach’s basic idea is that the student network updates its knowledge by distilling the information learned from the outputs of multiple teacher networks (soft labels) instead of segmenting using only target labels. The general flow diagram of the proposed method is shown in Figure 1.

In our approach, three different teacher networks are trained to learn different tasks. The first of these three teacher networks aims to segment the original targets (ground truths) corresponding to the input images. The second teacher network aims to segment only the thick vessels extracted from targets corresponding to the input images. The third teacher network aims to segment only the thin vessels extracted from targets corresponding to the input images. For four different datasets (DRIVE, CHUAC, CHASEDB1 and DCA1), thick and thin vessels in targets are manually annotated. For each dataset used in the experimental studies, the input images, the manually generated thin and thick ground truths, the original ground truths, and all the code developed in the study can be accessed from the GitHub link.

### Calculation of distillation loss and student loss

In this section, we describe the steps of the multi-teacher KD, focusing on the calculation of distillation loss and student loss. The overall objective is to train a student model that can effectively learn from multiple teacher models. The distillation loss measures how much the output distributions of the teacher and student networks differ. We employ Kullback–Leibler (KL) Divergence [28] as the primary metric for this purpose. Prior to calculating KL divergence, both the teacher and student logits are converted into probability distributions through the sigmoid activation function (Equation 1). This transformation is essential as it maps logits to a range of [0, 1], facilitating a meaningful comparison.

1 $$\begin{aligned} P_{\text {student}} = \sigma (z_{\text {student}}), \quad P_{\text {teacher}} = \sigma \left( \frac{z_{\text {teacher}}}{T}\right) \end{aligned}$$

where $\sigma (x) = \frac{1}{1 + e^{-x}}$ is the sigmoid function, $z_{\text {student}}$ and $z_{\text {teacher}}$ are the logits from the student and teacher models, respectively, and $T > 0$ is the temperature parameter. An increased value of $T$ results in smoother probability distributions, enhancing generalization [29]. The logits from the teacher network are adjusted using a temperature parameter $T$. This adjustment softens the output probabilities, enhancing gradient flow during training. The temperature parameter *T* plays a critical role in the distillation process by controlling the smoothness of the teacher’s probability distribution. We experimentally select the temperature parameter $T$ based on validation performance. The impact of various $T$ values on model performance is thoroughly analyzed in the ablation study outlined in Section"Temperature Tuning", where the results are discussed. This parameter helps achieve a balance between effective knowledge transfer and generalization. A small epsilon value is used to clamp probabilities, preventing issues with logarithm calculations when probabilities approach zero. To prevent numerical instability caused by $\log (0)$, probabilities are clamped within the range $[\epsilon , 1 - \epsilon ]$ defined in Equation 2, where $\epsilon = 10^{-7}$:

2 $$\begin{aligned} P_{\text {student}} \in [\epsilon , 1-\epsilon ], \quad P_{\text {teacher}} \in [\epsilon , 1-\epsilon ] \end{aligned}$$

The KL Deviation between the probabilities of the teacher and the student is calculated as shown in Equation 3.

3 $$\begin{aligned} \begin{aligned} \text {KL}(P_{\text {teacher}} \parallel P_{\text {student}}) = P_{\text {teacher}} \cdot \log \left( \frac{P_{\text {teacher}}}{P_{\text {student}}}\right) +\\ (1 - P_{\text {teacher}}) \cdot \log \left( \frac{1 - P_{\text {teacher}}}{1 - P_{\text {student}}}\right) \end{aligned} \end{aligned}$$

In the next step, the distillation loss of all teacher models needs to be calculated. Distillation loss is calculated individually for each teacher model (T1-original, T2-thick vessels, T3-thin vessels). The distillation loss from multiple teacher models is averaged across all models. For a multiple-teacher model, the distillation loss averaged across all teachers is calculated as in Equation 4,

4 $$\begin{aligned} L_{{avg}\_{distillation}}=\frac{1}{M}\sum _{k=1}^{M}L_{distillation}^{(k)} \end{aligned}$$

where $M$ is the number of teacher models, and $L_{\text {distillation}}^{(k)}$ represents the KL-Divergence loss for the $k$-th teacher. We have effectively trained the student model by using a combination of distillation loss and Binary Cross-Entropy (BCE) loss. The BCE loss evaluates the agreement between the student model and the true labels, whereas the distillation loss enables the student model to learn from the teacher model’s predictions. To integrate both losses, we introduce a weighting factor $\alpha$, which balances their contributions. The final loss for the student is a weighted sum of the distillation loss and the binary cross-entropy loss. The overall student loss $L_{student}$ is computed as shown in Equation 5.

5 $$\begin{aligned} L_{\text {student}} = \alpha \cdot L_{\text {avg}\_\text {distillation}} + (1 - \alpha ) \cdot L_{\text {BCE}} \end{aligned}$$

where $\alpha \in [0, 1]$ is a hyper-parameter that balances the contribution of the distillation loss and the BCE loss (In this study, the alpha value is selected as 0.5 for the weighted calculation of BCE loss and multi-teacher loss). This approach enables us to modify the impact of each loss type on the student’s learning process.

In summary, our method combines distillation and student losses through a carefully balanced approach, facilitating effective knowledge transfer from multiple specialized teachers to a single student model. By utilizing KL divergence for distillation and BCE for direct label comparison, our approach aims to enhance performance in RVS tasks.

## Experimental design

This section offers an overview of the datasets utilized in the study, details the process for creating the multi-teacher KD application, and introduces the evaluation metrics applied to measure the results.

### Datasets

In our experimental studies, four different vessel segmentation datasets are used. This section examines the features of each dataset separately and explains how the datasets are adapted to this study. In the experimental studies conducted with all datasets, no pre-processing is performed, and data augmentation is not implemented during the experimental studies.

The DRIVE dataset [30] includes 40 retinal images, which comprise both training and testing data. Corresponding target images are supplied in binary format and annotated by experts for every image. We have used the first 20 samples for training and the remaining for testing, following the train-test split defined by the authors [7, 30, 31]. The last two images of the 20 training images, along with their corresponding target images, are used for validation. The samples in the DRIVE are $565\times 584$ pixels and consist of three color channels. The labeled ground truths are based on the markings of the first observer.

The CHASEDB1 dataset consists 28 color retina images and their target images in binary format [32]. Each image is a 3-channel color channel with $999\times 960$ pixels. The first 20 images in the dataset are used for training, while the other 8 images are selected for testing. (as mentioned in reference studies) [7, 31].

The CHUAC dataset contains 30 single-channel coronary angiography images with $189\times 189$ pixels [32]. Experts manually created target images in a binary image format of $512\times 512$ pixels for each image in this dataset. The inputs are resized to $512\times 512$ pixels to ensure consistency in dimensions between the input and target images. Similar to prior research, this study utilizes the first 20 images for training and the last 10 images for testing testing [31, 33].

The DCA1 [34] dataset contains 134 X-ray coronary angiograms, each measuring $300\times 300$ pixels, along with the target images. The images are encoded in grayscale using the PGM image format and divided into two groups: training and testing. The training dataset contains 100 images, whereas the testing dataset has 34 images [7, 31].

**Fig. 2:**
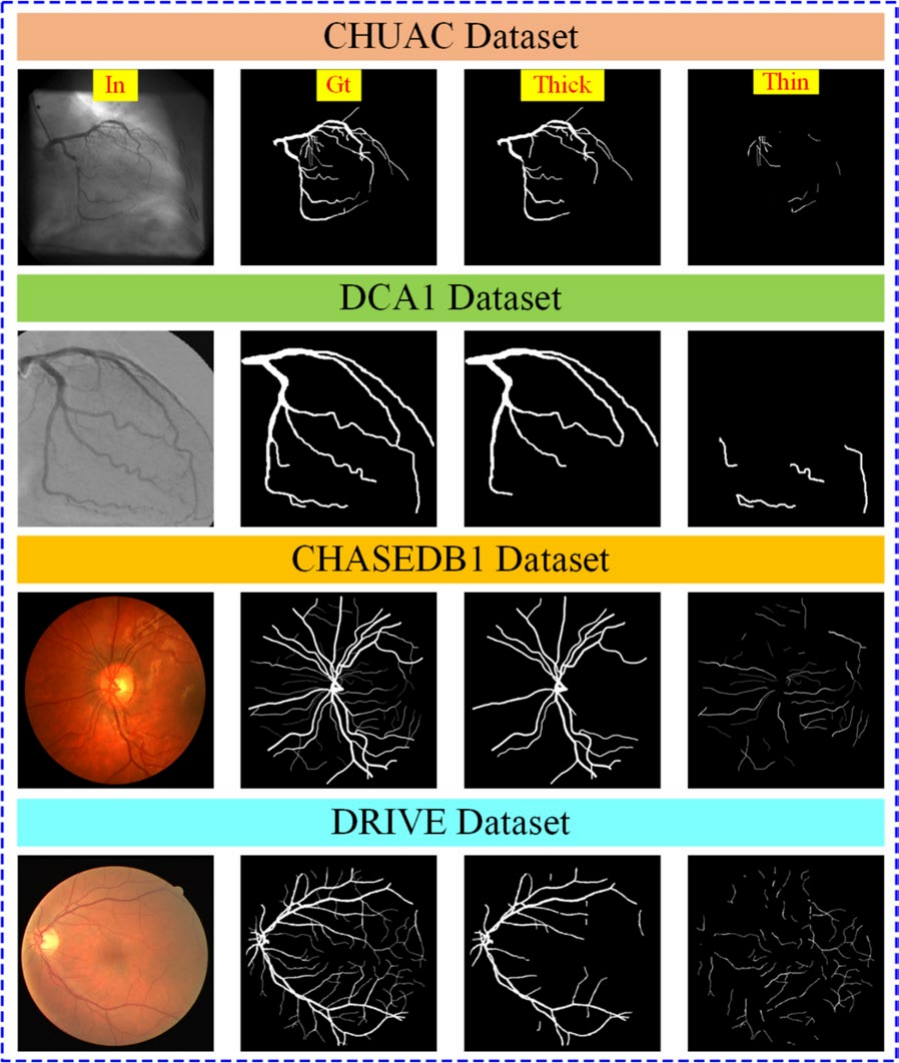
Datasets overview, In = input images, Gt = corresponding ground truth images, thick = manually labeled images consisting of only thick vessels, thin = Manually labeled images consisting of only thin vessels

Figure 2 shows sample images for four different datasets (DRIVE, CHUAC, CHASEDB1, and DCA1). The ground truth images for each image are manually labeled to include only thick and thin vessels.

### Implementation details

The training and testing phases are conducted systematically for each dataset, with a clear separation of training, validation, and test sets. In the DRIVE dataset, the first 18 samples are selected for training, while 2 samples are used for validation, and the last 20 samples are used for testing. Likewise, the CHASEDB1 dataset uses 18 samples for training, 2 for validation, and 8 samples for testing. For the CHUAC dataset, 16 samples are chosen for training, 4 for validation, and 10 for testing. The DCA1 dataset involves 80 samples for training, with 20 set aside for validation and 34 selected for testing. Validation samples for all datasets are separated from the training data, and test data remains untouched until the testing phase to ensure unbiased evaluation. During the training phase, three different models are initially trained for each dataset: T1 (original vessels model), T2 (thick vessels model), and T3 (thin vessels model). We have used the reported U-Net structure in [2] for T1, T2, and T3. These models served as teacher models, with"T"referring to"Teacher."After training, the best-performing T1, T2, and T3 models for each dataset are selected based on their validation performance. These top teacher models are then used to train a student model for each dataset using a multi-teacher KD method. In the testing phase, the performance of the student models is evaluated, and the best-performing student model for each dataset is used.

During the training of the models on each dataset, padding is applied to ensure size compatibility due to the dimensional differences among the datasets. We have applied 11$\times$8 pixels (11 pixels at the bottom and 8 pixels on the right), 20$\times$20 and 9$\times$48 paddings for DRIVE, DCA1 and CHASEDB1 datasets, respectively. On the other hand, for the CHASEDB1 dataset with an input size of 512$\times$512, no padding is required, as its dimensions are already compatible with the model’s input requirements.

We have selected the following hyper-parameters for common experimental studies. ReLU is used as the activation function in convolution layers, and batch normalization is performed before the activation function. The loss between the values predicted by the model and the targets is calculated with DiceBCELoss. Adam algorithm is used as the optimization method, and the learning rate is selected as $lr = 1e-3$. The model is trained for 250 training epochs with a batch size of = 8. Early stopping is not applied in model training; the model is tested with validation data at the end of each epoch, and the best model is saved based on validation loss.

The training and testing phases are conducted on a high-performance computer equipped with an NVIDIA RTX A5000 graphics card (24 GB GPU memory), an Intel Xeon Gold 6226R 2.90 GHz processor, and 128 GB of RAM. The source code and annotated thin, thick vessels are published on GitHub to ensure reproducibility.

### Evaluation metrics

In this study, Accuracy (ACC), Sensitivity (SEN), Specificity (SPE), F1-Score (F1), and Intersection over Union (IoU) are utilized as evaluation metrics. ACC measures the overall correctness of classifications, while SEN (Recall) is chosen to evaluate how well positive classes (e.g., vessel pixels) are detected. Conversely, SPE evaluates the correct classification rate of negative classes (e.g., non-vessel pixels), ensuring a minimal false-positive rate. F1 is employed to balance Precision and Recall. Intersection over Union (IoU) is used as a robust metric to quantify the overlap between segmentation outputs and ground truth. These metrics provide a well-rounded evaluation framework, ensuring the assessment of both overall performance and class balance. They are particularly critical for RVS tasks, where precision and sensitivity in distinguishing fine structures are crucial.

## Results and discussion

### Multi-teacher based knowledge distillation

The experimental study on Multi-Teacher-Based Knowledge Distillation (MTKD) aims to evaluate the proposed method’s performance against state-of-the-art architectures across four datasets: DRIVE, CHASEDB1, CHUAC, and DCA1. The evaluation metrics include accuracy (ACC), sensitivity (SEN), specificity (SPE), F1-score (F1), and intersection over union (IOU). The proposed method, MTKD-UNet (ours), leverages multiple teacher networks, each specialized in learning different vessel characteristics (original, thin, and thick vessels), to enhance the student network’s segmentation performance. These results are reported in Tables 1 and  2.

**Table 1:** Multi-teacher KD performance comparison of methods on different datasets-DRIVE and CHASEDB1

Dataset	Methods	Year	ACC	SEN	SPE	F1	IOU
DRIVE	U-Net [2]	2015	96.78	80.57	98.33	81.41	68.64
	UNET++ [9]	2018	96.79	78.91	98.50	81.14	68.27
	Attention U-Net [10]	2018	96.62	79.06	98.31	80.39	67.21
	CS-Net [12]	2019	96.32	81.70	98.54	82.65	70.43
	AG-Net [11]	2019	96.92	81.00	98.48	-	69.65
	RVSeg-Net [35]	2020	98.81	81.07	98.45	-	-
	SCS-Net [5]	2021	96.97	82.89	98.38	-	-
	SGL [6]	2021	97.05	83.80	98.34	83.16	-
	FR-UNet [7]	2022	97.05	83.56	98.37	83.16	71.20
	MTKD-UNet (ours)	2025	96.72	80.22	98.32	80.74	67.82
	MTKD-UNet (oursLP)	2025	96.93	79.54	98.60	81.66	69.08
CHASEDB1	U-Net [2]	2015	97.43	76.50	98.84	78.98	65.26
	UNET++ [9]	2018	97.39	83.57	98.32	80.15	66.88
	Attention U-Net [10]	2018	97.30	83.84	98.20	79.64	66.17
	CS-Net [12]	2019	97.42	84.00	98.32	80.42	67.25
	AG-Net [11]	2020	97.43	81.86	98.48	-	66.69
	RVSeg-Net [35]	2021	97.26	80.69	98.36	-	-
	SCS-Net [5]	2022	97.44	83.65	98.39	-	-
	SGL [6]	2021	97.71	86.90	98.43	82.71	-
	FR-UNet [7]	2022	98.48	87.98	98.14	81.51	68.82
	MTKD-UNet (ours)	2025	97.26	74.90	98.76	77.48	63.28
	MTKD-UNet (oursLP)	2025	97.54	78.09	98.83	79.95	66.62

The proposed MTKD-UNet achieves competitive results across all metrics on the DRIVE dataset. Specifically, it attains an accuracy of 96.72, sensitivity of 80.22, specificity of 98.32, F1-score of 80.74, and IOU of 67.82. While these results are slightly lower than those of some state-of-the-art methods like FR-UNet (ACC: 97.05, SEN: 83.56, F1: 83.16, IOU: 71.20) and SGL (SEN: 83.80, F1: 83.16), they are comparable to those of other methods such as U-Net and UNET++. The proposed method demonstrates strong specificity, indicating its ability to correctly identify non-vessel pixels, which is crucial for reducing false positives in medical imaging tasks.

For the CHASEDB1 dataset, MTKD-UNet achieves an accuracy of 97.26, sensitivity of 74.90, specificity of 98.76, F1-score of 77.48, and IOU of 63.28. While the sensitivity is lower compared to methods like FR-UNet (SEN: 87.98) and SGL (SEN: 86.90), the specificity is among the second highest, indicating robust performance in identifying non-vessel regions. The F1-score and IOU are also competitive, suggesting that the proposed method effectively balances precision and recall, which is critical for accurate vessel segmentation.

**Table 2:** Multi-teacher KD performance comparison of methods on different datasets-CHUAC and DCA1

Dataset	Methods	Year	ACC	SEN	SPE	F1	IOU
CHUAC	U-Net [2]	2015	97.84	58.81	99.40	67.68	51.15
	UNET++ [9]	2018	98.12	66.87	99.37	73.23	57.77
	Attention U-Net [10]	2018	98.00	65.26	99.13	71.54	55.69
	CS-Net [12]	2019	97.96	67.35	99.18	71.71	55.89
	FR-UNet [7]	2022	98.03	81.71	98.68	76.01	61.51
	MTKD-UNet (ours)	2025	98.23	73.33	99.40	76.02	61.41
	MTKD-UNet (oursLP)	2025	98.19	75.34	99.10	76.12	61.57
DCA1	U-Net [2]	2015	97.58	78.16	98.66	77.35	63.07
	UNET++ [9]	2018	97.61	79.54	98.62	77.86	63.75
	Attention U-Net [10]	2018	97.55	79.86	98.53	77.48	63.24
	CS-Net [12]	2019	97.63	78.95	98.67	77.90	63.80
	FR-UNet [7]	2022	97.88	82.48	97.75	80.22	67.08
	MTKD-UNet (ours)	2025	97.84	80.19	98.84	79.38	66.0
	MTKD-UNet (oursLP)	2025	97.79	84.48	98.54	79.87	66.63

On the CHUAC dataset, the proposed method achieves the highest accuracy (98.23) and specificity (99.40) among all methods, outperforming even FR-UNet (ACC: 98.03, SPE: 98.68) and U-Net (SPE: 99.40). The sensitivity (73.33) and F1-score (76.02) are also competitive, though slightly lower than FR-UNet (SEN: 81.71, F1: 76.01). The high specificity and accuracy suggest that the proposed method is particularly effective in handling the CHUAC dataset, which may have unique characteristics that benefit from the multi-teacher approach.

For the DCA1 dataset, MTKD-UNet (ours) achieves an accuracy of 97.84, sensitivity of 80.19, specificity of 98.84, F1-score of 79.38, and IOU of 66.0. The specificity is the highest among all methods, indicating excellent performance in identifying non-vessel regions. The sensitivity and F1-score are competitive, though slightly lower than FR-UNet (SEN: 82.48, F1: 80.22). The proposed method’s ability to achieve high specificity while maintaining reasonable sensitivity and F1-score highlights its robustness in handling diverse datasets. Our method uniquely employs three teacher networks, each specialized in segmenting different vessel types: original, thin, and thick vessels. This specialization allows the student network to distill rich, complementary knowledge that captures vessel heterogeneity more effectively than single-teacher or non-specialized methods. This targeted distillation enhances the student’s ability to segment vessels of varying thicknesses, especially improving the challenging thin vessel segmentation, which is a known limitation in many prior works. Compared to classical U-Net and its variants (e.g., Attention U-Net), our method achieves higher Dice and IoU scores by effectively leveraging multi-scale vessel information through knowledge distillation. While these methods rely on single-network learning, they often struggle with thin vessel detection.

### Multi-teacher based KD with penalization

**Fig. 3:**
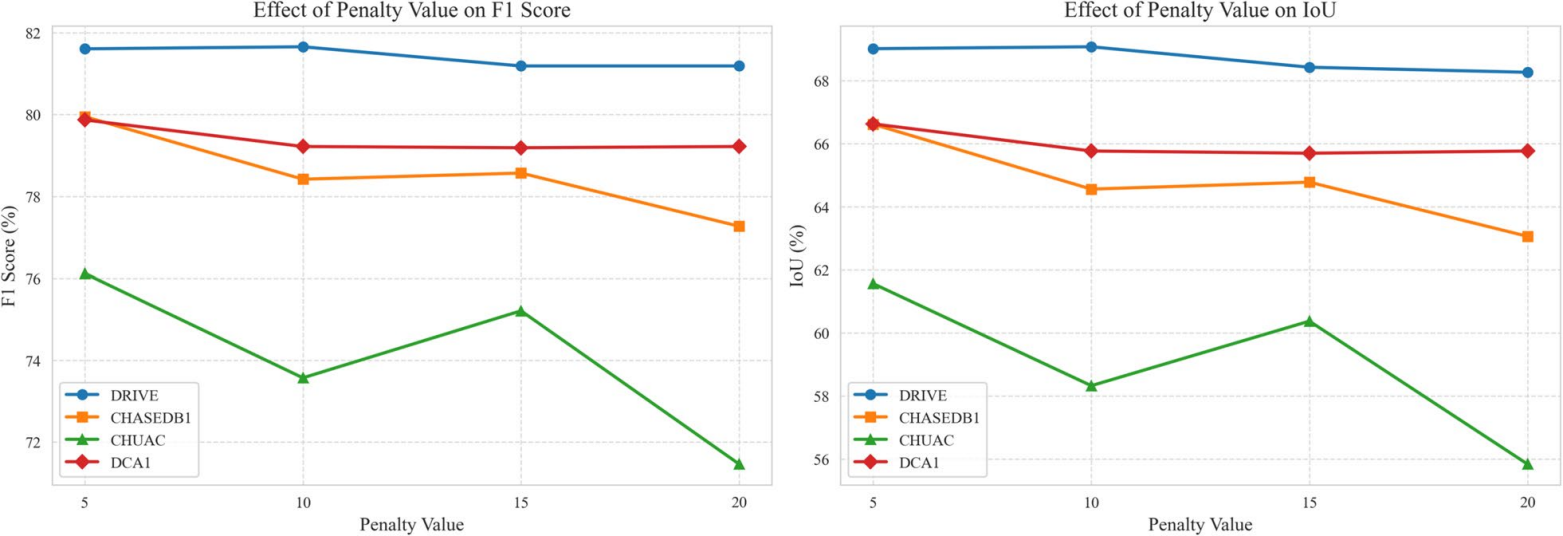
Effect of penalty on model performance for DRIVE, CHASEDB1, CHUAC and DCA1 datasets

This experiment applies a penalization technique to the student model’s loss function to enhance segmentation performance. This involves multiplying the loss obtained from the student network by a penalty factor. This approach emphasizes the importance of the student’s loss during training, potentially leading to more accurate segmentation. We have systematically explored penalty values of 5, 10, 15, and 20, incrementing by 5 to observe the effect on model performance. Our experimental results showed that performance improvements plateaued and, in some cases, slightly decreased when penalty values exceeded 20 (see Figure 3). This observation guided our decision to limit the exploration to this range. Specifically, a penalty of 10 is found to be best for the DRIVE dataset, while a penalty of 5 is used for the other datasets (CHASEDB1, CHUAC, and DCA1).

On the DRIVE dataset, the penalized method, MTKD-UNet (oursLP), achieves an accuracy of 96.93, sensitivity of 79.54, specificity of 98.60, F1-score of 81.66, and IOU of 69.08. Compared to the non-penalized version, MTKD-UNet (ours) (ACC: 96.72, SEN: 80.22, SPE: 98.32, F1: 80.74, IOU: 67.82), the penalized method shows an improvement in accuracy (+0.21 points), specificity (+0.28 points), F1-score (+0.92 points), and IOU (+1.26 points). However, sensitivity decreased slightly ($-$0.68 percentage points). While the penalized method showed competitive results compared to state-of-the-art methods like FR-UNet (accuracy: 97.05%, sensitivity: 83.56%, F1-score: 83.16%, IOU: 71.20%) and SGL (sensitivity: 83.80%, F1-score: 83.16%), it did not outperform them. The high specificity indicates that the penalized method effectively reduces false positives.

For the CHASEDB1 dataset, MTKD-UNet (oursLP) achieves an accuracy of 97.54, sensitivity of 78.09, specificity of 98.83, F1-score of 79.95, and IOU of 66.62. Compared to the non-penalized version, MTKD-UNet (ours) (ACC: 97.26, SEN: 74.90, SPE: 98.76, F1: 77.48, IOU: 63.28), the penalized method shows improvements in all metrics: accuracy (+0.28 points), sensitivity (+3.19 points), specificity (+0.07 points), F1-score (+2.47 points), and IOU (+3.34 points). This demonstrates that penalization significantly enhances the model’s ability to segment both thin and thick vessels, particularly improving sensitivity and F1-score. Compared to state-of-the-art methods, MTKD-UNet (oursLP) does not surpass FR-UNet (ACC: 98.48, SEN: 87.98, F1: 81.51, IOU: 68.82) or SGL (SEN: 86.90, F1: 82.71). However, it achieves competitive specificity, outperforming FR-UNet by 0.69 points and SGL by 0.40 points. This indicates that the penalized method is robust in identifying non-vessel regions, even if it lags slightly in sensitivity and F1-score.

On the CHUAC dataset, the penalized method achieves an accuracy of 98.19, sensitivity of 75.34, specificity of 99.10, F1-score of 76.12, and IOU of 61.57. In comparison, our non-penalized version, MTKD-UNet, records an accuracy of 98.23, sensitivity of 73.33, specificity of 99.40, F1-score of 76.02, and IOU of 61.41. The penalized method shows a slight decrease in accuracy (by 0.04 points) and specificity (by 0.30 points), while it achieves improvements in sensitivity (by 2.01 points), F1-score (by 0.10 points), and IOU (by 0.16 points). This indicates that penalization enhances the model’s ability to detect thin vessels (sensitivity) and overall segmentation performance (F1 and IOU), even if it slightly reduces specificity. Notably, MTKD-UNet (oursLP) outperforms FR-UNet by 0.11 percentage points in terms of F1-score and by 0.06 percentage points in IOU. Additionally, it surpasses other state-of-the-art methods regarding F1-score and IOU, suggesting a good balance between precision and recall.

For the DCA1 dataset, MTKD-UNet (oursLP) achieves an accuracy of 97.79, sensitivity of 84.48, specificity of 98.54, F1-score of 79.87, and IOU of 66.63. In comparison, the non-penalized version, MTKD-UNet (ours), shows an accuracy of 97.84%, sensitivity of 80.19%, specificity of 98.84%, F1-score of 79.38%, and IoU of 66.0%. The penalized version shows a slight decrease in accuracy (by 0.05 points) and specificity (by 0.30 points), but improvements are seen in sensitivity (by 4.29 points), F1-score (by 0.49 points), and IoU (by 0.63 points). This indicates that penalization enhances the model’s ability to detect thin vessels (sensitivity) and overall segmentation performance (F1 and IOU), even if it slightly reduces specificity. When compared to state-of-the-art methods, our MTKD-UNet (oursLP) model demonstrates competitive results, particularly in sensitivity, where it outperforms FR-UNet (sensitivity: 82.48%) by 2.00 points. While the F1-score and IoU are also competitive, they are slightly lower than those of FR-UNet. The high specificity of the penalized method indicates its effectiveness in reducing false positives, which is crucial for accurate segmentation in medical imaging.

In conclusion, MTKD-UNet (oursLP) demonstrates competitive performance across multiple datasets, particularly in terms of specificity and accuracy. The penalized version further improves sensitivity, making it a strong candidate for applications where detecting thin vessels is critical. Additionally, it generally performs better than the non-penalized version, MTKD-UNet (ours), across all datasets. The penalization technique appears to improve the student model’s learning process, resulting in better segmentation outcomes. Overall, the proposed methods surpass the baseline U-Net model in most metrics. However, the penalized method does not consistently outperform state-of-the-art methods like FR-UNet or SGL. Applying this LP technique results in distinct performance differences between the MTKD-UNet (oursLP) and the non-penalized MTKD-UNet (ours) versions. Consistently across several datasets, the LP variant demonstrates improved sensitivity, which the authors interpret as an enhanced capability to detect finer, thinner vessels. This improvement likely stems from the increased weight given to the ground truth loss, compelling the model to minimize errors even on challenging positive examples (thin vessels). However, this heightened focus on capturing positive pixels sometimes leads to a slight decrease in specificity. This suggests an inherent trade-off: the model, by becoming more aggressive at identifying vessel pixels (improving sensitivity), may occasionally misclassify background pixels as vessels, thereby slightly increasing the false positive rate and reducing specificity. Despite this potential minor decrease in specificity, the results generally show that the F1-score and Intersection over Union (IOU) improve with the LP technique. This indicates that the benefit gained from correctly identifying more vessel pixels (higher sensitivity) typically outweighs the negative impact of potentially incorrectly classifying slightly more background pixels.

### Ablation studies

The ablation studies conducted in this study aim to systematically evaluate the impact of various components and configurations on the performance of the proposed method for RVS. These experiments are designed to provide insights into the effectiveness of different loss functions and the balance between model complexity and performance. They also examine the individual contributions of teacher models, the sensitivity of the student model’s architecture, and the influence of the temperature parameter on KD. The findings from these ablation studies not only validate the design choices of the proposed framework but also offer valuable guidance for future research in KD and RVS.

#### Selection of the loss function

This ablation study examines how different loss functions influence the performance of the student model during KD. While the distillation loss is fixed, the study evaluates the impact of various loss functions applied to the student network on the overall knowledge transfer and segmentation performance. The primary objective of this ablation study is to analyze the influence of different loss functions on the student model’s learning process and its ability to generalize from the soft labels provided by the teacher models. We aim to determine which loss components contribute most significantly to the student’s F1 score and Intersection over Union (IoU) performance by varying the loss function.

The experiment includes training the student model with eight different loss functions: DiceLoss, CombinedLoss, TverskyLoss, ComboLoss, SoftDiceLoss, DiceBCELoss, FocalLoss, and FocalTverskyLoss. The distillation loss function remains unchanged, ensuring that the comparison focuses solely on the impact of the student’s loss function. The performance metrics are evaluated on four datasets: DRIVE, CHASEDB1, CHUAC, and DCA1, as reported in Table 3.

As shown in Table 3, the results show that the choice of loss function significantly impacts the student model’s performance. For the DRIVE dataset, the ComboLoss achieves the highest F1 score (81.61) and IoU (69.02), indicating that a combination of Dice and cross-entropy losses provides the best balance between precision and recall. Similarly, for the CHASEDB1 dataset, the CombinedLoss yields the best F1 score (79.95) and IoU (66.62), suggesting that combining multiple loss functions can enhance the student’s ability to learn from the teacher models.

**Table 3:** Performance comparison of different loss functions on student network

	DRIVE		CHASEDB1		CHUAC		DCA1	
Loss function	F1	IOU	F1	IOU	F1	IOU	F1	IOU
DiceLoss	81.32	68.68	78.98	65.32	74.52	59.54	78.95	65.41
CombinedLoss	81.56	68.93	**79**.**95**	**66**.**62**	75.7	61.08	79.43	66.03
TverskyLoss	81.24	68.52	78.56	64.75	73.43	58.18	79.35	65.94
ComboLoss	**81**.**61**	**69**.**02**	79.02	65.36	75.27	60.45	79.12	65.61
SoftDiceLoss	80.90	68.08	78.34	64.46	74.71	59.8	79.16	65.66
DiceBCELoss	81.11	68.29	78.09	64.15	**76**.**12**	**61**.**57**	**79**.**87**	**66**.**63**
FocalLoss	81.12	68.31	75.71	61.12	72.41	56.91	78.28	64.5
FocalTverskyLoss	81.54	68.91	78.11	64.14	74.69	59.72	79.34	65.91

The bold values indicate the best results for each metric and dataset

For the CHUAC dataset, the DiceBCELoss achieves the highest F1 score (76.12) and IoU (61.57), demonstrating that a combination of Dice and binary cross-entropy losses is particularly effective for this dataset. The results indicate that a loss function that prioritizes pixel-wise accuracy and class balance improves the CHUAC dataset. For the DCA1 dataset, the DiceBCELoss again yields the best performance, achieving an F1 score of 79.87 and an IoU of 66.63. This further confirms its effectiveness across various datasets.

Interestingly, the FocalLoss and FocalTverskyLoss underperform compared to other loss functions, particularly on the CHUAC and DCA1 datasets. This suggests that while focal losses are designed to address class imbalance, they may not be as effective in KD, where the soft labels already provide a balanced representation of the teacher models’ predictions.

The ablation study demonstrates that the choice of loss function for the student network is a critical factor in KD. The ComboLoss and CombinedLoss consistently perform well across multiple datasets, indicating that combining multiple loss components can enhance the student’s ability to perform overall knowledge transfer and segmentation performance. The DiceBCELoss also performs well, particularly on angiography datasets. These findings suggest that future work could explore adaptive loss functions to optimize distillation.

#### Impacts of complexity in student network

This ablation study aims to analyze the trade-off between the size of the student model and its performance in RVS tasks. The objective is to determine whether a smaller, more efficient student model (referred to as the"Lite Student") can achieve comparable performance to the original, larger student model while significantly reducing computational complexity. This study is especially important for applications in resource-constrained environments, such as mobile devices or real-time medical imaging systems, where computational efficiency is critical.

In this study, we have compared the performance of the original student model, based on the standard U-Net architecture, with a Lite student model, where the number of feature maps in each layer is halved. The comparison is conducted across four datasets: DRIVE, CHASEDB1, CHUAC, and DCA1. The metrics used for evaluation included SEN, SPE, F1, and IOU (see Table 4). Additionally, we have compared the computational efficiency of the two models in terms of the number of parameters (#Params), number of FLOPs (#Flops), and parameter size (see Table 5).

The results revealed a clear trade-off between model size and performance. On the DRIVE dataset, the Lite student model showed a slight drop in performance, with the sensitivity (SEN) decreasing by 0.82 (from 79.54 to 78.72) and specificity (SPE) falling by 0.42 (from 98.60 to 98.18). The F1-Score decreased by 2.22 (from 81.66 to 79.44), and the IOU decreased by 3.17 (from 69.08 to 65.91). On the CHASEDB1 dataset, the Lite student model performed slightly better in terms of sensitivity (+2.78, from 78.09 to 80.87) but showed a minor drop in specificity ($-$0.38, from 98.83 to 98.45). The F1-Score decreased by 0.74 (from 79.95 to 79.21), and the IOU decreased by 0.99 (from 66.62 to 65.63). However, on the CHUAC and DCA1 datasets, the Lite student model struggled, with significant drops in sensitivity ($-$4.69 for DCA1). The F1-Score decreased by 2.97 and 3.73, respectively, and the IOU decreased by 3.76 and 4.99, respectively. This suggests that reducing the model size may not always be advantageous, especially for datasets with more complex vessel structures.

**Table 4:** Performance Comparison of Student and Lite Student Models on DRIVE. CHASEDB1. CHUAC. and DCA1 Datasets. $\triangle$ indicates the difference between Student and Lite Student networks

	DRIVE				CHASEDB1			
Model	**SEN**	**SPE**	**F1**	**IOU**	**SEN**	**SPE**	**F1**	**IOU**
Student	79.54	98.60	81.66	69.08	78.09	98.83	79.95	66.62
Lite Student	78.72	98.18	79.44	65.91	80.87	98.45	79.21	65.63
$\triangle$	$-$0.82	$-$0.42	$-$2.22	$-$3.17	2.78	$-$0.38	$-$0.74	$-$0.99

	CHUAC				DCA1			
	**SEN**	**SPE**	**F1**	**IOU**	**SEN**	**SPE**	**F1**	**IOU**
Student	75.34	99.1	76.12	61.57	84.48	98.54	79.87	66.63
Lite Student	76.08	98.73	73.15	57.81	79.79	98.41	76.14	61.64
$\triangle$	0.74	$-$0.37	$-$2.97	$-$3.76	$-$4.69	$-$0.13	$-$3.73	$-$4.99

In terms of computational efficiency, the Lite student model demonstrated significant improvements. For example, on the DRIVE dataset, the number of parameters has reduced from 31.04M to 0.487M, the number of FLOPs has reduced from 284.35G to 4.53G, and the parameter size has reduced from 124.17MB to 1.95MB. Similar reductions are observed across the other datasets, making the Lite student model highly efficient in terms of computational resources. The number of parameters and parameter size for the other datasets decreased similarly. However, the number of FLOPs is reduced for CHASEDB1, CHUAC, and DCA1 to 65, 16, and 85 times, respectively.

Our analysis also explores how dataset characteristics contribute to performance differences with a simplified student network. We examine factors such as image resolution, vessel tortuosity and thickness, contrast, noise levels, and imaging modalities across the DRIVE, CHASEDB1, CHUAC, and DCA1 datasets. For example, datasets with complex, low-contrast, or finer-caliber vessels (like CHUAC and DCA1) tend to degrade the performance of a lightweight model, which struggles to learn detailed features. Conversely, datasets with relatively more uniform and precise vessel representations might exhibit less performance drop. We highlight that DRIVE and CHASEDB1 comprise retinal fundus images, while CHUAC and DCA1 come from Coronary Angiography (CA). This difference significantly influences performance, as CA images face challenges like lower signal-to-noise ratios, dynamic contrast flow, and vessel morphology variations compared to the relatively static and well-defined structures in fundus images. Consequently, the"Lite Student"model, with its halved feature map capacity, may struggle more significantly with the inherent complexities and potentially greater variability within CA datasets like CHUAC and DCA1. Moreover, we analyze how the Lite model’s reduced capacity affects its feature capture ability. Interestingly, its sensitivity can improve in some cases (e.g., CHASEDB1, CHUAC) even as F1 and IoU decrease, possibly indicating a trade-off where the model learns generalized features that enhance recall but reduce precision in specific datasets.

In conclusion, this ablation study highlights the trade-off between model size and performance. While the Lite student model is computationally efficient, it may not always achieve the same level of performance as the original student model, particularly on more complex datasets. The Lite student model is a viable option for applications where computational efficiency is critical. However, the original student model may be preferable for tasks requiring high accuracy. Future work could focus on further optimizing the Lite student model using advanced compression techniques or architecture search to bridge the performance gap while maintaining efficiency.

**Table 5:** Comparison of the computational complexities of the Student and Lite Student networks on RVS datasets. The number of parameters and the parameter size are reported in megabytes, and the number of floating-point operations per second (FLOPs) is reported in gigabytes

Dataset	Model	#Params (M)	#Flops (G)	Parameter Size (MB)
**DRIVE**	Student	31.04	284.35	124.17
	Lite Student	0.487	4.53	1.95
**CHASEDB1**	Student	31.04	847.28	124.17
	Lite Student	0.487	13.51	1.95
**CHUAC**	Student	31.04	218.30	124.17
	Lite Student	0.487	13.36	1.95
**DCA1**	Student	31.04	85.27	124.17
	Lite Student	0.487	1.35	1.95

#### Teacher Model Contribution Analysis

This ablation study investigates the individual contributions of each teacher model within the proposed Multi-Teacher Knowledge Distillation (MTKD) method for RVS. The primary objective is to determine whether combining multiple teachers provides complementary knowledge to the student network or if a single teacher dominates the learning process. This is crucial for understanding the efficiency of the multi-teacher approach and identifying potential redundancies or imbalances in the knowledge transfer process.

**Fig. 4:**
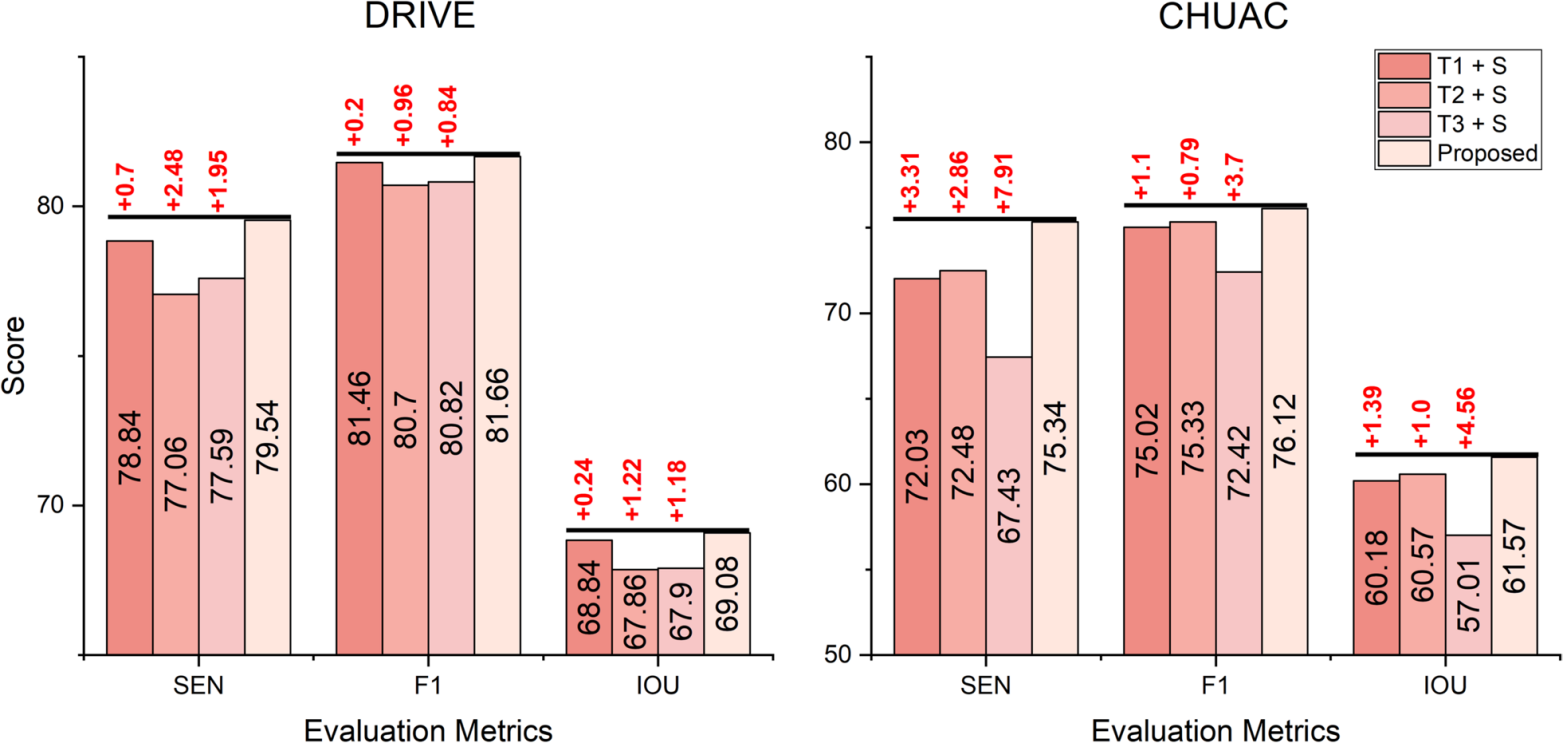
Evaluation of individual teacher contributions to student model performance: Comparison of single-teacher training (T1+S, T2+S, T3+S) to Multi-Teacher setup across evaluation metrics (SEN, F1, IOU) for DRIVE and CHUAC datasets. T1, T2, and T3 represent teacher models trained with original, thick, and thin ground truths. The text highlighted in red illustrates the differences between the proposed method and other approaches

The experimental design involves training the student network using only one teacher at a time. Specifically, three separate training runs are conducted, each utilizing a single teacher model: Teacher 1 (T1) is trained with the original ground truth; Teacher 2 (T2) is trained with thick vessels; and Teacher 3 (T3) is trained with thin vessels. The performance of the student network trained with each individual teacher is then compared to the performance of the student network trained with all three teachers (the proposed MTKD approach). The evaluation metrics used for comparison are SEN, F1 and IOU, which are reported for the DRIVE and CHUAC datasets in Figure 4.

The results presented in Figure 4 indicate that the proposed multi-teacher approach consistently outperforms single-teacher configurations across all metrics and datasets. For the DRIVE dataset, the proposed method achieves a sensitivity of 79.54, an F1-score of 81.66, and an IOU of 69.08. These values are higher than those obtained when training with individual teachers. Specifically, the sensitivity is improved by 0.7, 2.48, and 1.95 when compared to T1+S, T2+S, and T3+S, respectively. Similarly, the F1-score shows improvements of 0.2, 0.96, and 0.84, and the IOU is enhanced by 0.24, 1.22, and 1.18 for T1+S, T2+S, and T3+S, respectively. The CHUAC dataset also shows superior performance with the proposed method, achieving a sensitivity of 75.34, an F1-score of 76.12, and an IOU of 61.57. These results are improved by 3.31, 2.86, and 7.91 for SEN, 1.1, 0.79, and 3.7 for F1, and 1.39, 1.0, and 4.56 for IOU compared to T1+S, T2+S, and T3+S, respectively.

These findings suggest that the multi-teacher approach provides complementary knowledge to the student network. Each teacher learns about different parts of the vessel structure, such as the original, thin, and thick parts, and shares this unique knowledge with the student. This helps the student to better identify and segment retinal vessels. We interpret this outcome as evidence that each teacher develops unique expertise, having been trained on a specific aspect of the vessel structure (overall, thick or thin parts). Combined, these specialized teachers share their distinct knowledge with the student model. For instance, the teacher trained on thin vessels becomes adept at identifying those fine structures, while the teacher trained on thick vessels excels at segmenting the larger ones, and the ’original’ teacher provides a general overview. This collaborative approach generates synergy, as the student model benefits from this diverse set of perspectives, allowing it to integrate specialized knowledge from each teacher to attain a more comprehensive and accurate understanding of the entire retinal vasculature. We conclude that no single teacher dominates the learning process, and the combination is essential for optimal performance, highlighting the effectiveness of leveraging the diverse expertise encapsulated within the multiple teachers. This underscores the effectiveness of the proposed MTKD framework in utilizing the diverse expertise of multiple teachers to improve the robustness and accuracy of RVS.

#### Student Model Architecture Sensitivity

The ablation study on Student Model Architecture Sensitivity explains how varying the student model’s architecture impacts the KD process. The architecture of the student model is a critical factor in determining its ability to learn effectively from teacher models. This study evaluates the performance of five different architectures–ResNet-18, ResNet-50, MobileNet v2, EfficientNet, and DenseNet-121–on the DRIVE and CHUAC datasets, using SEN, F1, and IoU as evaluation metrics. We have used U-Net as a base segmentation architecture and only changed the encoder/decoder networks.

**Fig. 5:**
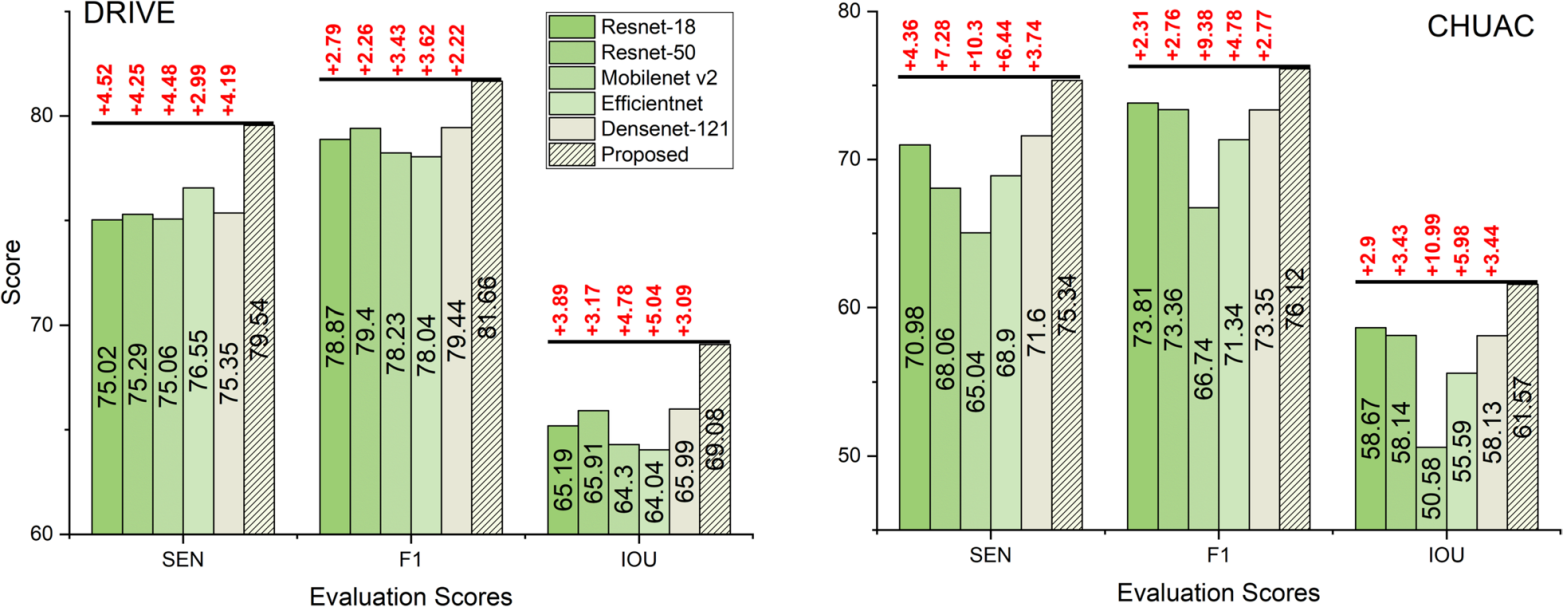
Evaluation of the student architecture sensitivity with different encoder/decoder networks across evaluation metrics (SEN, F1, IOU) for DRIVE and CHUAC datasets. The text highlighted in red illustrates the differences between the proposed method and others

The results presented in Figure 5 indicate that the choice of student model architecture significantly impacts performance. On the DRIVE dataset, the proposed architecture achieves the highest F1 score (81.66) and IoU (69.08), outperforming all other architectures. The differences between the proposed and other architectures can be up to 4.52 points for SEN, 3.62 points for F1, and 5.04 points for IOU. It also demonstrates strong sensitivity (79.54), indicating its ability to accurately detect true positives. Among the alternative architectures, DenseNet-121 and ResNet-50 perform relatively well, with F1 scores of 79.44 and 79.40, IoUs of 65.99 and 65.91, and sensitivities of 83.12 and 82.95, respectively. These results suggest that deeper architectures or those with dense connections are more effective in capturing the complex features required for RVS. In contrast, lightweight architectures such as MobileNet v2 and EfficientNet show lower performance, with F1 scores of 78.23 and 78.04, IoUs of 64.30 and 64.04, and sensitivities of 81.45 and 81.20, respectively. This indicates that while these architectures are computationally efficient, they may not have the capacity to capture the complex details of retinal vessels fully.

The proposed model on the CHUAC dataset demonstrates significant improvements over the other architectures. It achieves the highest sensitivity (75.34), outperforming ResNet-18 by 4.36 points and MobileNet v2 by 10.3 points. For the F1 score, the proposed model achieves 76.12, with DenseNet-121 being the closest competitor at 73.35, a difference of 2.77 points. MobileNet v2 again shows the largest gap, with an F1 score 9.38 points lower than the proposed model. The IoU metric follows a similar trend, with the proposed model achieving 61.57, outperforming DenseNet-121 by 3.44 points and MobileNet v2 by 10.99 points. This further highlights the limitations of lightweight architectures in handling complex segmentation tasks.

Architectures like DenseNet-121 and ResNet-50 also perform relatively well, while lightweight architectures such as MobileNet v2 and EfficientNet show lower performance. They are more effective in capturing the complex features required for RVS. Conversely, it suggests that lightweight architectures, despite their computational efficiency, may not have the capacity to capture the complex details of retinal vessels fully. The study also highlights the importance of architectural compatibility between teacher and student models. We note that student models with architectures more similar in nature or capacity to the teacher models tend to perform better. Student models with architectures similar to the teacher models, such as ResNet-18 and ResNet-50, generally perform better than those with different architectures, such as MobileNet v2 and EfficientNet. This suggests that architectural similarity enables better knowledge transfer during the distillation process. Therefore, the explanation is that architectures with sufficient capacity (like ResNet and DenseNet) are better suited for the inherent complexity of the RVS task. They are also better for effectively receiving and integrating the distilled knowledge from the similarly complex U-Net-based teacher ensemble, facilitated by this architectural compatibility.

#### Temperature Tuning

In this ablation study, we aim to investigate the impact of the temperature parameter (*T*) on the performance of the student model. The temperature parameter plays a critical role in KD by controlling the smoothness of the soft labels generated by the teacher models. A higher temperature produces softer probability distributions, which can help the student model generalize better. On the other hand, a lower temperature results in sharper distributions, which may lead to overfitting.

The primary objective of this ablation study is to analyze how varying the temperature parameter affects the student model’s performance across different datasets. By tuning the temperature, we aim to identify an optimal value that maximizes the transfer of knowledge from the teacher models to the student model. This improvement is expected to boost sensitivity, specificity, F1 score, and Intersection over Union.

The experiment includes training the student model with different temperature values (*T* = 2.0, 3.0, 4.0, 5.0, 7.0) while keeping other hyperparameters fixed. We have selected these temperature values based on insights from Cho and Hariharan [21] and recent studies on knowledge distillation [28, 36], and systematically evaluated temperature values ranging from 2.0 to 7.0. The performance metrics (Sensitivity, Specificity, F1 score, and IoU) are evaluated on the DRIVE and CHUAC datasets, as reported in Table 6. The goal is to observe how the temperature parameter influences the student model’s ability to learn from the soft labels provided by the teacher models.

**Table 6:** Performance of Multi-Teacher knowledge distillation with varying temperature values. The bold values indicate the best results for each metric and dataset

	DRIVE				CHUAC			
	SEN	SPE	F1	IOU	SEN	SPE	F1	IOU
**T = 2,0**	79,36	98,58	81,36	68,76	73,94	**99,17**	75,91	61,28
**T = 3,0**	79,59	98,59	**81,61**	**69,02**	75,34	99,1	**76,12**	**61,57**
**T = 4,0**	**80,00**	98,23	80,32	67,19	**84,83**	99,11	75,64	60,94
**T = 5,0**	79,71	98,48	81,13	68,40	74,17	98,83	72,80	57,35
**T = 7,0**	75,37	**98,96**	80,56	67,66	68,67	99,02	70,65	54,91

**Fig. 6:**
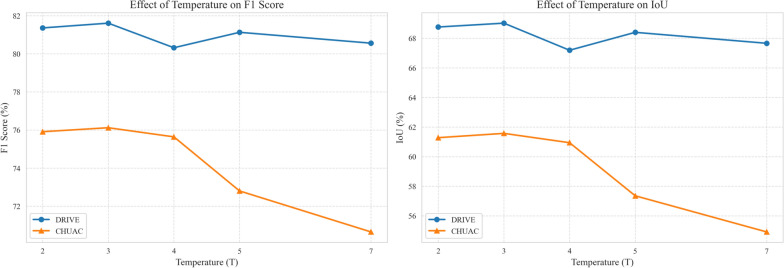
Effect of temperature on model performance for DRIVE and CHUAC datasets

The results presented in Table 6 highlight the significant impact of the temperature parameter on the performance of the student model. Furthermore, we have presented a comprehensive figure (Figure 6) illustrating how F1 score and IoU metrics change with different temperature settings across our datasets. For the DRIVE dataset, the best performance is achieved at *T*=3.0, with an F1 score of 81.61 and an IoU of 69.02. This indicates that a moderate temperature value provides an optimal balance between soft label transfer and the student’s learning capability. In contrast, higher temperatures ($T = 4.0$, $T = 5.0$, and $T = 7.0$) lead to decreased performance, suggesting that excessively soft labels can impede the student’s ability to learn distinctive features.

For the CHUAC dataset, the optimal temperature is also *T* = 3.0, yielding the highest F1 score (76.12) and IoU (61.57). Interestingly, at *T* = 4.0, the sensitivity peaks at 84.83. However, this comes at the cost of a lower F1 score and IoU, This suggests that while the model detects more true positives, it may also increase the number of false positives, thereby reducing the overall effectiveness.

The ablation study demonstrates that the temperature parameter is a critical factor in KD. A temperature of *T* = 3.0 consistently provides the best balance between soft label transfer and the student model’s learning efficiency across both datasets. This finding aligns with the theoretical understanding that moderate temperatures improve generalization by smoothing probability distributions without significantly reducing discriminative information. Future work could explore adaptive temperature scheduling or dataset-specific tuning to further optimize the distillation process.

## Conclusion

This study introduces a novel Multi-Teacher Based Knowledge Distillation (MTKD) framework for RVS, addressing the challenge of accurately segmenting both thin and thick vessels in retinal images. The proposed framework utilizes the expertise of multiple teacher networks, each specialized in learning distinct vessel characteristics–such as original, thin, and thick vessels–and transfers this diverse knowledge to a single student network. This approach significantly enhances the segmentation of thin vessels, which are often overlooked by existing methods, while maintaining high accuracy for thick vessels. The framework’s ability to generalize across diverse datasets and its competitive performance compared to state-of-the-art methods make it a notable advancement in the field.

This work makes many key contributions. First, the Response-Based Multi-Teacher Knowledge Distillation framework aggregates knowledge from specialized teacher models and transfers it to a unified student network, improving its generalization capabilities. Second, the method is rigorously evaluated on four datasets (DRIVE, CHASEDB1, CHUAC, and DCA1), demonstrating highly competitive performance. Third, extensive ablation studies are conducted to analyze the impact of various components, such as loss functions, model complexity, teacher contributions, and temperature tuning, providing valuable insights into the framework’s effectiveness. Additionally, a loss penalization technique is introduced to the student model’s loss function, further enhancing segmentation performance by emphasizing the importance of the student’s loss during training. Finally, all source codes, datasets, and manually annotated ground truths were made publicly available to support reproducibility and encourage further research.

The results highlight that the proposed MTKD framework outperforms traditional U-Net-based approaches and achieves state-of-the-art performance on certain datasets. However, while the method excels in specificity and accuracy, it occasionally lags behind in sensitivity compared to advanced methods like FR-UNet and SGL. T his suggests that while the framework is highly effective in reducing false positives, there is room for improvement in detecting true positives, particularly in complex datasets. Our extensive ablation studies provide critical insights into the dynamics of knowledge transfer within this framework. The identified optimal loss functions (like DiceBCELoss and ComboLoss) highlight the necessity of balancing pixel-level accuracy with regional overlap agreement to effectively translate teacher knowledge into robust student segmentation. Similarly, the optimal distillation temperature (*T*=3.0) represents a carefully calibrated equilibrium, softening teacher outputs sufficiently to convey generalizable patterns without obscuring the fine-grained details essential for accurate RVS. The effectiveness of the student models with architectures similar to the teacher models further suggests that architectural compatibility is vital for efficiently absorbing the distilled multi-faceted knowledge. Additionally, the inherent multi-scale processing capabilities of encoder-decoder structures play an essential role in this process. Furthermore, the introduced loss penalization technique proved crucial; its success is interpreted as a necessary mechanism to ground the student’s learning, preventing over-reliance on potentially smoothed teacher outputs and ensuring focused optimization towards the primary segmentation goal [19, 20].

In future works, feature-based KD could be investigated to transfer intermediate feature representations from teacher models to the student, potentially enhancing segmentation performance. Additionally, adaptive loss functions that dynamically adjust based on dataset complexity or vessel characteristics could be developed. Architecture optimization techniques, such as advanced compression or neural architecture search, could also be explored to balance computational efficiency and segmentation accuracy.

## Data Availability

All codes and data used in this study are open source and can be downloaded from the link below. https://github.com/msaaydin/MTKD-Multi-Teacher-Based-Knowledge-Distillation
